# Early evidence of delayed oligodendrocyte maturation in the mouse model of mucolipidosis type IV

**DOI:** 10.1242/dmm.044230

**Published:** 2020-07-30

**Authors:** Molly Mepyans, Livia Andrzejczuk, Jahree Sosa, Sierra Smith, Shawn Herron, Samantha DeRosa, Susan A. Slaugenhaupt, Albert Misko, Yulia Grishchuk, Kirill Kiselyov

**Affiliations:** 1 Center for Genomic Medicine and Department of Neurology, Massachusetts General Hospital Research Institute and Harvard Medical School, Boston, MA 02114, USA; 2 Department of Biological Sciences, University of Pittsburgh, Pittsburgh, PA 15260, USA

**Keywords:** Mucolipidosis type IV, Lysosome, TRPML1, Mucolipin-1, Myelination, Oligodendrocyte

## Abstract

Mucolipidosis type IV (MLIV) is a lysosomal disease caused by mutations in the *MCOLN1* gene that encodes the endolysosomal transient receptor potential channel mucolipin-1, or TRPML1. MLIV results in developmental delay, motor and cognitive impairments, and vision loss. Brain abnormalities include thinning and malformation of the corpus callosum, white-matter abnormalities, accumulation of undegraded intracellular ‘storage’ material and cerebellar atrophy in older patients. Identification of the early events in the MLIV course is key to understanding the disease and deploying therapies. The *Mcoln1*^−/−^ mouse model reproduces all major aspects of the human disease. We have previously reported hypomyelination in the MLIV mouse brain. Here, we investigated the onset of hypomyelination and compared oligodendrocyte maturation between the cortex/forebrain and cerebellum. We found significant delays in expression of mature oligodendrocyte markers *Mag*, *Mbp* and *Mobp* in the *Mcoln1^−/−^* cortex, manifesting as early as 10 days after birth and persisting later in life. Such delays were less pronounced in the cerebellum. Despite our previous finding of diminished accumulation of the ferritin-bound iron in the *Mcoln1*^−/−^ brain, we report no significant changes in expression of the cytosolic iron reporters, suggesting that iron-handling deficits in MLIV occur in the lysosomes and do not involve broad iron deficiency. These data demonstrate very early deficits of oligodendrocyte maturation and critical regional differences in myelination between the forebrain and cerebellum in the mouse model of MLIV. Furthermore, they establish quantitative readouts of the MLIV impact on early brain development, useful to gauge efficacy in pre-clinical trials.

## Abstract (precis)

**Summary:** We show that loss of the lysosomal channel TRPML1, responsible for mucolipidosis IV, leads to delayed maturation of oligodendrocytes in early postnatal development, resulting in brain hypomyelination.

## INTRODUCTION

Mucolipidosis type IV (MLIV) is an autosomal-recessive lysosomal storage disease caused by mutations in the *MCOLN1* gene, located in humans in the 19p13.2 locus (Acierno et al., 2001). The disease was first described in 1974 (Berman et al., 1974) and the corresponding gene was reported in 1999 (Bargal et al., 2000; Raas-Rothschild et al., 1999; Slaugenhaupt et al., 1999). *MCOLN1* encodes the late endosomal/lysosomal ion channel TRPML1, which has a range of reported functions including zinc and iron homeostasis, regulation of lysosomal acidification and calcium release driving membrane fusion/fission events, as well as lysosomal biogenesis (Cheng et al., 2014; Dong et al., 2008; Eichelsdoerfer et al., 2010; Kukic et al., 2013; Miao et al., 2015; Samie et al., 2013; Soyombo et al., 2006; Venkatachalam et al., 2008; Wong et al., 2012; Xu and Ren, 2015; Xu et al., 2006; Zhang et al., 2012). Abnormalities of membrane traffic, autophagy and mitochondria have been reported in various MLIV tissues and models (Coblentz et al., 2014; Colletti et al., 2012; Jennings et al., 2006; Li et al., 2016; Miedel et al., 2008; Venkatachalam et al., 2008; Vergarajauregui et al., 2008; Wong et al., 2012; Xu et al., 2006; Zhang et al., 2016).

MLIV patients typically present in the first year of life with psychomotor delay and visual impairment, which stems from a combination of corneal clouding, retinal dystrophy and optic atrophy (Abraham et al., 1985; Altarescu et al., 2002; Riedel et al., 1985; Schiffmann et al., 2014). Developmental gains can be appreciated during the first decade of life, although axial hypotonia, appendicular hypertonia and upper motor neuron weakness prevent ambulation and limit fine motor function in the majority of patients. Although a static course has been classically reported, progressive psychomotor decline has been documented. It begins in the second decade, eventually resulting in severe spastic quadriplegia (A.M., personal observations). In congruence with the clinical course, ancillary brain imaging has demonstrated stable white-matter abnormalities (corpus callosum hypoplasia/dysgenesis and white-matter lesions), with the emergence of subcortical volume loss and cerebellar atrophy in older patients. Despite a 19-year-long effort in the MLIV natural history program (NCT00015782, NCT01067742), owing to the low number of patients and poor availability of the human brain tissue for research (single case tissues are now available at the University of Maryland Brain and Tissue Bank, Baltimore, MD, USA), the mechanism(s) of MLIV brain disease is still not well understood. Pinpointing the early events in MLIV brain pathology is important to define the therapeutic window for intervention and set expectations for clinical trial outcomes.

Some important insights regarding brain pathology in MLIV have been obtained from the MLIV mouse model [*Mcoln1* knockout (KO) mouse] developed by us (Grishchuk et al., 2014, 2016; Micsenyi et al., 2009; Venugopal et al., 2007). The mouse model shows all the hallmarks of MLIV, including motor deficits, retinal degeneration and reduced secretion of chloric acid by stomach parietal cells, leading to elevated plasma gastrin (Schiffmann et al., 1998). These hallmarks were also supported by the murine MLIV model generated by the Muallem group (Chandra et al., 2011). Similar to MLIV patients, we observed intracellular storage formations, thinning of the corpus callosum in the *Mcoln1*^−/−^ mouse brain, micro- and astrogliosis, and partial loss of Purkinje cells at the late stage of the disease. Electron microscopy analysis shows reduced thickness of the myelin sheaths consistent with central nervous system (CNS) hypomyelination (Grishchuk et al., 2014).

Based on the existing natural history data in MLIV patients and our observations in the *Mcoln1^−/−^* mouse, MLIV can be thought of as having two distinct phases: the developmental phase, associated with early-onset delayed oligodendrocyte maturation and gliosis (Grishchuk et al., 2014; Weinstock et al., 2018), and the late degenerative phase, associated with partial loss of Purkinje cells in the MLIV mouse (Micsenyi et al., 2009) and mild cerebellar atrophy in some MLIV patients (Frei et al., 1998). Understanding the timing and mechanisms behind these distinct events is important for designing and testing therapies. Here, we investigate the role of TRPML1 in oligodendrocyte maturation and myelin deposition in the developing brain. Our data show early problems with oligodendrocyte maturation and myelination in higher-functioning brain regions such as the cerebral cortex. The role of TRPML1 in early brain development is further supported by the fact that its expression gradually rises in postnatal brain and expression is higher in the regions that display the most prominent deficits in myelination in TRPML1-deficient (*Mcoln1^−/−^*) mice (cerebral cortex). Finally, these data establish quantitative readouts of the TRPML1 impact on early brain development, which will be useful to gauge efficacy readouts in future therapy trials.

## RESULTS

### *Mcoln1*/TRPML1expression increases with age in the postnatal brain

**Fig. 1.**
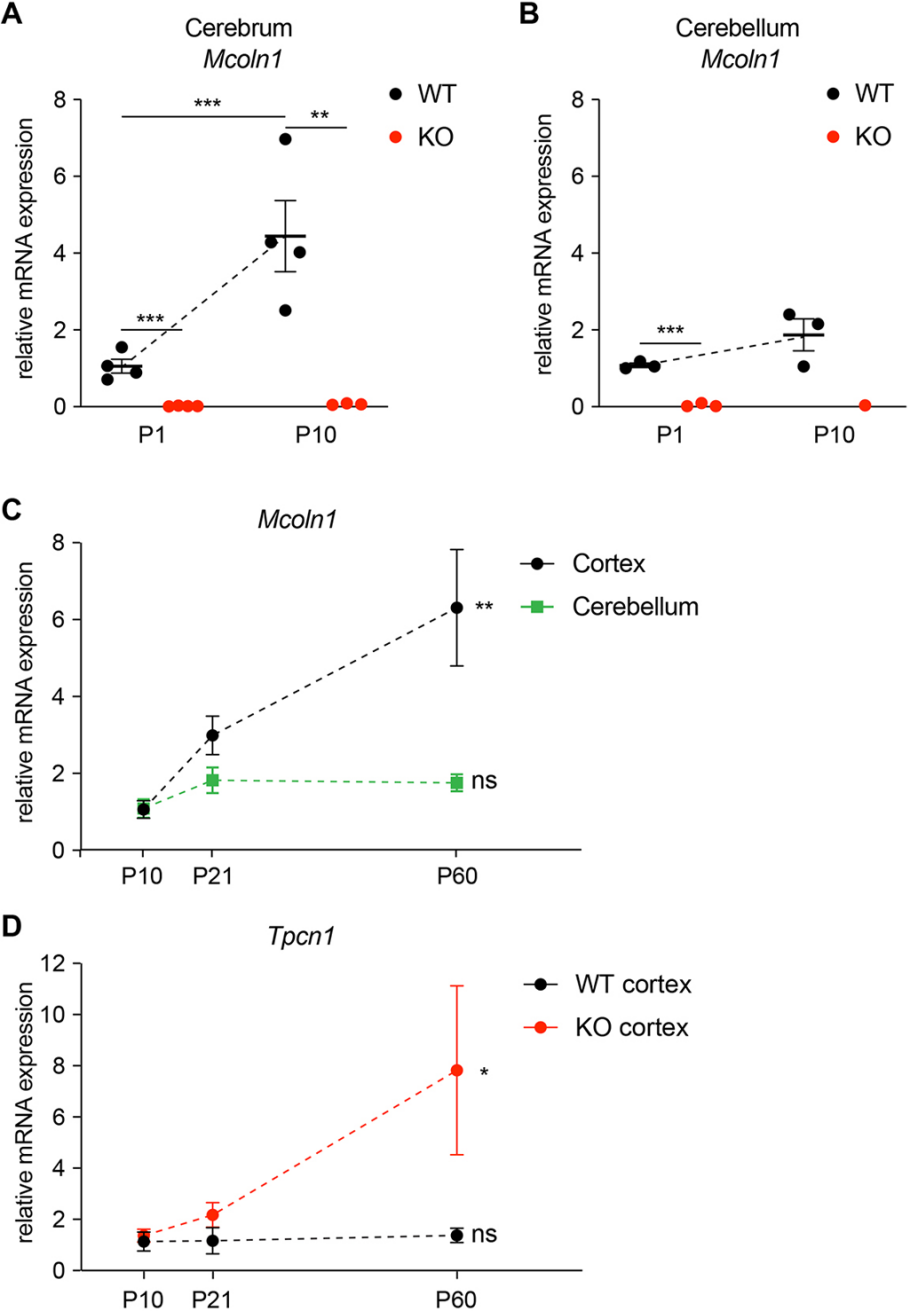
**Increasing *Mcoln1* expression in the brain during development.** (A,B) qPCR analysis of *Mcoln1* mRNA in the whole forebrain (A) and cerebellum (B) in *Mcoln1*^+/+^ (WT) and *Mcoln1*^−/−^ (KO) mice, showing a significant increase with age. Analysis of expression was performed using ΔΔ^Ct^ method; *Actb* was used as a housekeeping gene. Statistical analysis was performed using two-way ANOVA followed by Tukey's multiple comparison tests. *n*=3-5; ***P<0.0005 and **P<0.005. (C) *Mcoln1* expression dynamics in the cortical and cerebellum samples of P10-P60 mice. The symbols represent an average of three to four experiments. Three to four biological replicates; **P<0.005 calculated using one-way ANOVA. (D) *Tpcn1* mRNA expression in the *Mcoln1*^+/+^ and *Mcoln1*^−/−^ cerebral cortex. *n*=3-5; *P<0.05 calculated using one-way ANOVA. ns, *P*>0.05; error bars represent s.e.m.

Despite the emerging role of TRPML1 in a range of cellular functions, including cationic lysosomal homeostasis, cell repair (Colletti et al., 2012; Li et al., 2016; Miedel et al., 2008; Venkatachalam et al., 2008; Vergarajauregui et al., 2008; Wong et al., 2012; Xu et al., 2006; Zhang et al., 2016) and, more recently, aging and cancer (Jung et al., 2019; Xu et al., 2019), the time course of *Mcoln1*/TRPML1 expression during the development of living tissue has not been tracked. To answer how *Mcoln1*/TRPML1 is regulated during brain development we analyzed its expression using quantitative reverse transcription PCR (qRT-PCR) in 1-, 10-, 21- and 60-day-old mice. Actin B (*Actb*) was used as a housekeeping gene. Primer specificity was confirmed using *Mcoln1*^−/−^ samples. Our data show a steady increase in *Mcoln1* expression levels in the forebrain from birth to 2 months of age (Fig. 1). *Mcoln1* mRNA levels increased 4-fold (4.45±0.56, *n*=3 or 4, *P*=0.003) in the whole-cerebrum samples of wild-type (WT) animals between postnatal day (P)1 and P10 (Fig. 1A). Focusing specifically on the isolated cortical tissues, we found a 6-fold increase in *Mcoln1* mRNA levels between P10 and P60 (6.31±1.10, *n*=3, statistically significant linear trend with *P*=0.0028 using one-way ANOVA; Fig. 1C). By contrast, although *Mcoln1* mRNA levels also increased in the cerebellar samples between P1 and P10 (Fig. 1B) and P10 and P60 (Fig. 1C), the increases were small and did not attain statistical significance or significant linear trend. We found a similar trend in the *Mcoln1* expression data generated by the Functional Annotation of Mammalian Genome (FANTOM5) project (Ha et al., 2019; Lizio et al., 2015; Noguchi et al., 2017; Vitezic et al., 2014) (Fig. S1A). Although the design of the study does not allow direct comparison of transcriptional activation of *Mcoln1* between visual cortex and cerebellum, the stable expression of *Mcoln1* in cerebellum in the pre- and early-postnatal phase and increased expression in postnatal visual cortex are consistent with the data we report here. Our *Mcoln1* expression data are the first on the developmental dynamics of *Mcoln1* transcription that allow comparison between different brain regions, cortex and cerebellum.

*Mcoln1*-encoded endolysosomal cation channel TRPML1 is involved in lysosomal function and signaling (Xu and Ren, 2015). We compared its expression profile with that of another endolysosomal cation channel, TPC1 (encoded by *Tpcn1*) (Yamaguchi et al., 2011). Interestingly, *Tpcn1* mRNA did not increase with age in the mouse cerebral cortex (Fig. 1D). This, to our knowledge, is the first analysis of *Tpcn1* expression in the developing brain. Therefore, *Mcoln1* mRNA upregulation during development is specific to this gene rather than a more general increase in expression of the lysosomal channels. The difference between *Mcoln1* and *Tpcn1* expression implies that functions of the corresponding ion channels in the developing brain are distinct. The rapid pace of *Mcoln1* mRNA increase in the developing brain suggests its importance in development. Interestingly, there was a significant increase in the *Tpcn1* mRNA levels in the *Mcoln1*^−/−^ samples at the P60 time point (7.83±2.27-fold increase, *n*=3, statistically significant linear trend with *P*=0.047 using one-way ANOVA). RNA sequencing analysis on isolated brain cell types shows enrichment of the *Tpcn1* transcripts specifically in astrocytes, macrophage/microglia and endothelial cells (Zhang et al., 2014); therefore, the increase in its expression in *Mcoln1^−/−^* brain could reflect astrocytosis and microgliosis, a known prominent feature of brain pathology in MLIV (Grishchuk et al., 2014; Micsenyi et al., 2009; Weinstock et al., 2018) (Fig. S1B).

### Early myelination deficits due to loss of *Mcoln1*/TRPML1 are caused by delayed oligodendrocyte maturation

**Fig. 2.**
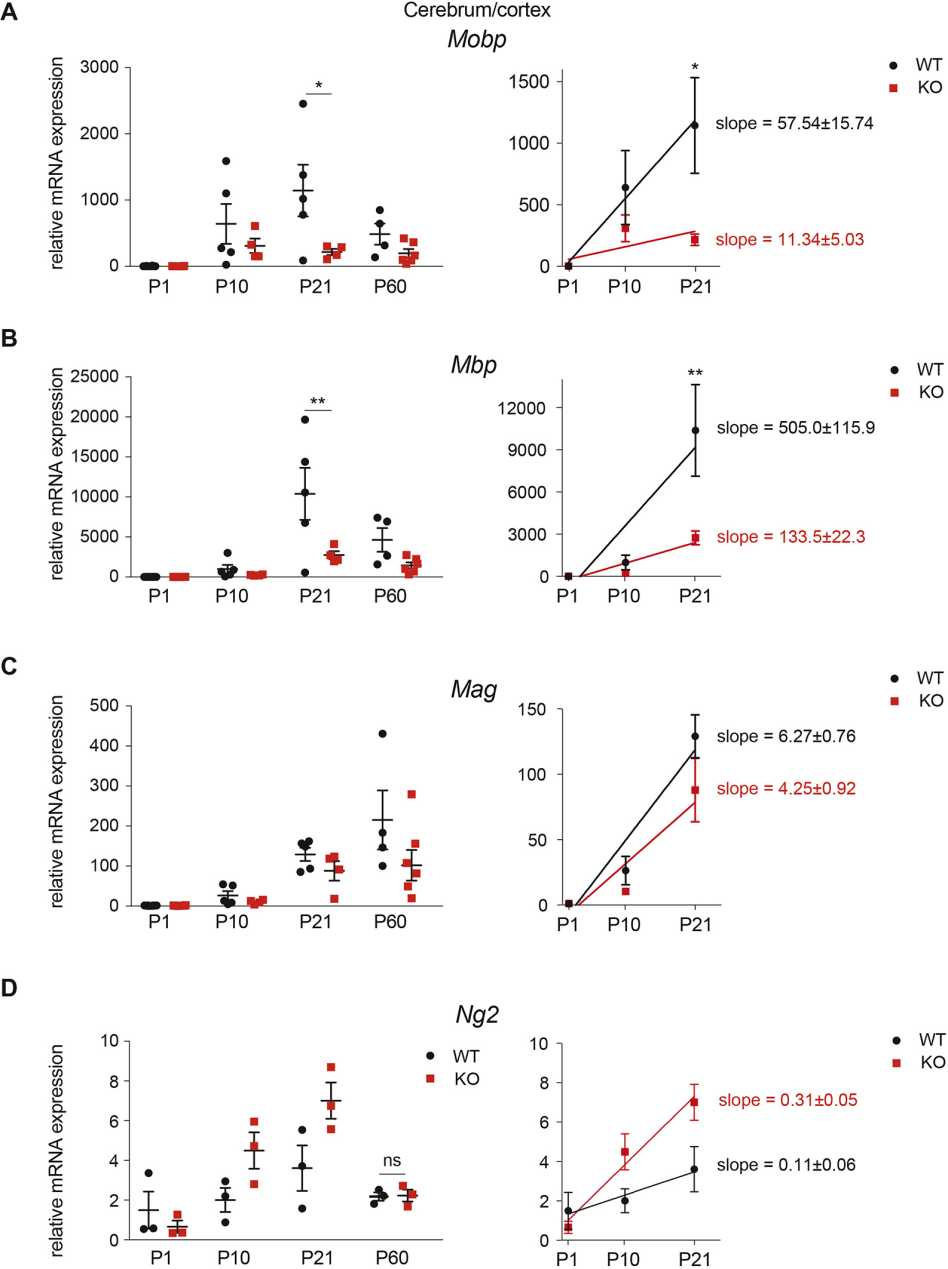
**Delayed oligodendrocyte maturation in the** ***Mcoln1*^−/−^ cerebral cortex.** (A-D) qPCR analysis of cerebrum (P1) and cortex (P10, P21 and P60) of *Mcoln1*^+/+^ and *Mcoln1*^−/−^ littermate mice, showing expression of the mature oligodendrocyte markers *Mobp*, *Mbp* and *Mag* (A-C) and oligodendrocyte precursor marker *Ng2* (D). The analysis was performed using the ΔΔ^Ct^ method; *Actb* was used as a housekeeping gene. Slopes were obtained using simple linear regression, all of which were significant except WT in D. **P<0.005 and *P<0.05 using two-way ANOVA followed by Tukey's multiple comparison tests; *n*=3-5; ns, *P*>0.05; error bars represent s.e.m.

We have previously shown hypomyelination in the mouse model of MLIV (Grishchuk et al., 2015), suggesting oligodendrocyte involvement. The original data, however, did not clarify whether the hypomyelination is a result of a lower number of oligodendrocytes due to loss of precursors or caused by their defective maturation. To gain more insight, we performed an expression time course of oligodendrocyte maturation markers in postnatal development starting at birth to 2 months of age. To assess oligodendrocyte maturation, we used three of the key myelin genes: myelin-associated glycoprotein precursor (*Mag*), myelin basic protein (*Mbp*) and myelin-associated oligodendrocyte basic protein (*Mobp*) (Han et al., 2013; Huang et al., 2013; Pérez et al., 2013; Robinson et al., 2014). In the forebrain samples, the three markers showed linear increase between P1 and P21, allowing us to compare the dynamics of oligodendrocyte maturation during brain development (Fig. 2). In both WT and KO samples, *Mobp* mRNA showed a statistically significant linear trend (*P*=0.0021 and *P*=0.0438, respectively, calculated using one-way ANOVA, *n*=3 or 4; Fig. 2A). At P21, *Mobp* mRNA increase was significantly higher in the *Mcoln1*^+/+^ relative to *Mcoln1*^−/−^ samples (*P*=0.037 using unpaired Student's *t*-test, *n*=5-7). This trend persisted for *Mbp* (Fig. 2B). Interestingly, while *Mag* mRNA levels showed linear increase between P1 and P21, the rate of *Mag* mRNA increase was significantly lower than that of *Mobp* and *Mbp* mRNA and did not differ significantly between *Mcoln1^+/+^* relative to *Mcoln1^−/−^* samples (Fig. 2C), suggesting that *Mcoln1* loss actuates specific changes in oligodendrocyte maturation rather than the loss of entire cell populations. The relative abundance of *Mbp* and *Mobp* mRNA decreased between P21 and P60 (Fig. 2A,B), whereas levels of *Mag* mRNA continued to grow (Fig. 2C). These data are compatible with other published results (Ehmann et al., 2013). The observation that the expression of all three maturation markers was lower in the *Mcoln1^−/−^* cortices compared with *Mcoln1^+/+^* cortices demonstrates early myelination delays (Fig. 2).

To answer whether the observed delays in the acquisition of the mature oligodendrocyte gene expression in the cortex are the result of delayed oligodendrocyte maturation or caused by loss of oligodendrocytes precursors, we analyzed the expression of the oligodendrocyte precursor marker neural/glial antigen 2 (*Ng2*; also known as *Cspg4*) (Hill et al., 2014). Interestingly, we found that expression of *Ng2* increased in the *Mcoln1^−/−^* cortices compared with *Mcoln1^+/+^* cortices (Fig. 2D). Increase in *Ng2* expression might reflect the attempt to overcome the downstream myelination deficits during the active phase of brain myelination in postnatal development. Therefore, we conclude that brain hypomyelination in MLIV is caused by delayed oligodendrocyte maturation and reduced myelin production rather than the death of the oligodendrocyte precursors.

**Fig. 3.**
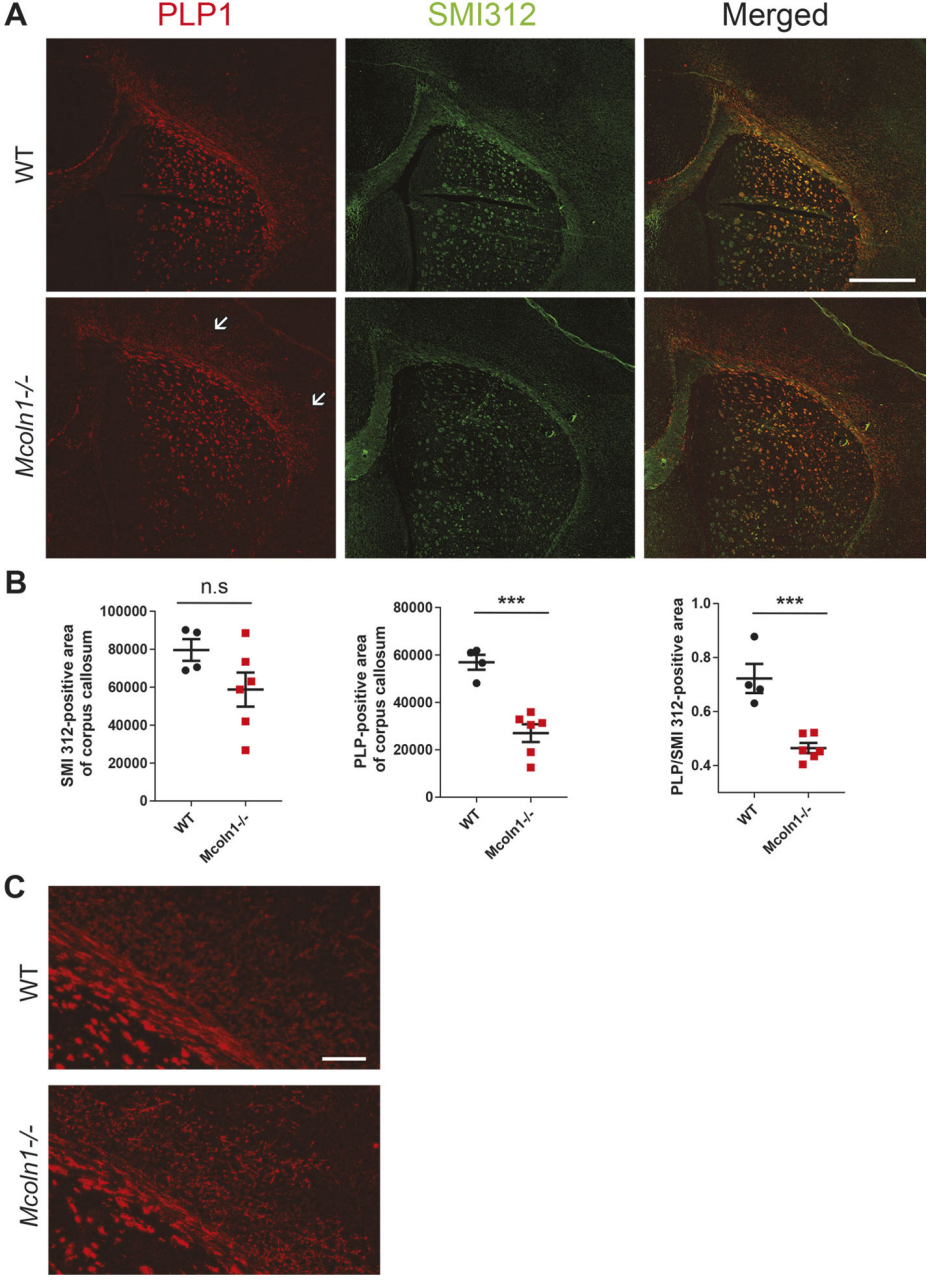
**Reduced myelination of the corpus callosum in the MLIV mouse model is not accompanied by significant reduction in neuronal fiber thickness.** (A) Representative images of *Mcoln1*^+/+^ and *Mcoln1*^−/−^ P10 brain sections stained with the mature oligodendrocyte/myelin marker PLP1 (red) and neuronal filament marker SMI312 (green). Corpus callosum and cortical areas are shown. Arrows show oligodendrocyte-deficient lesions in the *Mcoln1*^−/−^ brain. Scale bar: 1 μm. (B) Analysis of the corpus callosum area identified via either PLP1 or SMI312 staining, showing reduction of the PLP1-positive but not SMA312-positive area and significant reduction of the PLP1- to SMI312-positive area ratio in *Mcoln1*^−/−^ mice, indicating deficient myelination but normal underlying structure in the forming corpus callosum in the MLIV mouse. *n*=4 (*Mcoln1*^+/+^) and *n*=6 (*Mcoln1*^−/−^); ***P<0.001 using unpaired Student's *t*-test; n.s, *P*>0.05; error bars represent s.e.m. (C) Enlarged representative images of the *Mcoln1*^+/+^ and *Mcoln1*^−/−^ P10 brain sections immunostained for PLP1, showing dysmorphic myelinating fibers in the *Mcoln1*^−/−^ brain. Scale bar: 200 nm.

Next, we used immunohistochemistry to analyze developmental myelination of the corpus callosum in 10-day-old *Mcoln1*^+/+^ and *Mcoln1*^−/−^ mice using antibodies for the mature oligodendrocyte marker myelin proteolipid protein (PLP1) (Wang et al., 2006), one of the most abundant proteins in the myelin, and the neuronal neurofilament marker SMI312 (Vasung et al., 2011) (Fig. 3). Previously, we reported that thinning of the corpus callosum in 2- and 7-month-old *Mcoln1^−/−^* mice, while the thickness of the underlying neuronal fibers in the corpus callosum remained intact, was caused by defective myelination (Grishchuk et al., 2015). Similar to these observations made in adult mice, we here detect no significant decrease in the SMI312-positive area of the corpus callosum in P10 pups, representing the normally forming tract of the callosal neuronal fibers, but observed a significant decrease in myelin deposition by mature oligodendrocytes, demonstrated by reduced PLP1-positive area alone and decreased ratio of the PLP1-positive area to the corresponding SMI312-positive area (Fig. 3A,B). Furthermore, we observed the irregular distribution of PLP1-positive cells and forming myelinated fibers in the *Mcoln1*^−/−^ cortex, the islands of tissue that are devoid of the PLP1 staining (Fig. 3C and arrows in A), further supporting defective maturation of oligodendrocyte precursors in this model.

### Delayed maturation of oligodendrocytes in the *Mcoln1^−/−^* brain is region specific

**Fig. 4.**
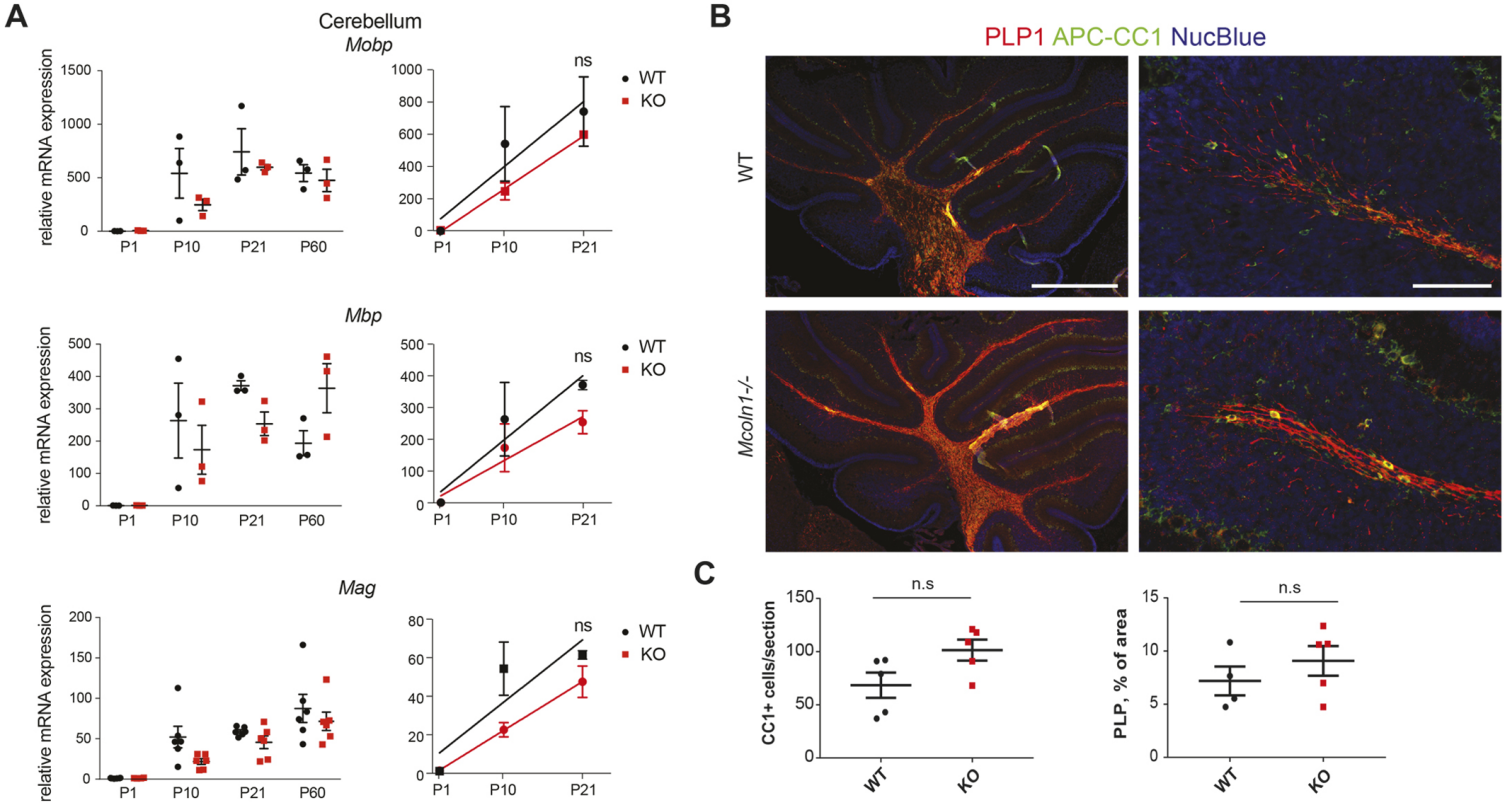
**Cerebellar myelination is preserved in MLIV mice.** (A) qPCR analysis of P1, P10, P21 and P60 cerebella from *Mcoln1*^+/+^ (WT) and *Mcoln1*^−/−^ (KO) littermate mice, showing expression of the mature oligodendrocyte markers *Mobp*, *Mbp* and *Mag*. The analysis was performed using the ΔΔ^Ct^ method; *Actb* was used as a housekeeping gene. ns, *P*>0.05 using two-way ANOVA followed by Tukey's multiple comparison test; *n*=3-5; error bars represent s.e.m. (B) Representative images of the *Mcoln1*^+/+^ and *Mcoln1*^−/−^ P10 brain sections stained with the mature oligodendrocyte/myelin marker PLP1 (red), oligodendrocyte marker APC-CC1 (green) and nuclear counterstain Nuc-Blue (blue). Scale bars: 500 nm (left), 100 nm (right). (C) Statistical analysis of the APC-CC1-positive cell counts per section (left) and PLP1-positive area (right) in the cerebellar sections show no significant changes between *Mcoln1*^+/+^ and *Mcoln1*^−/−^ mice. n.s, *P*>0.05 using unpaired Student's *t*-test; *n*=4 (*Mcoln1^+/+^*) and *n*=5 (*Mcoln1^−/−^*).

To answer whether the impact of *Mcoln1*/TRPML1 loss on myelination is different in the cerebellum, we performed quantitative PCR (qPCR) analysis on the cerebellar samples from P1-P60 mice. We found that the overall differences in expression of the mature oligodendrocyte maturation markers in *Mcoln1^−/−^* and control *Mcoln1^+/+^* cerebella were not as pronounced as they were in the cortical samples (Fig. 4A). This qPCR analysis was also supported by immunohistochemistry, using which we detected no difference in the percentage of PLP1-positive area, nor in the counts of oligodendrocytes labeled by APC-CC1 (Fig. 4B,C), in *Mcoln1^−/−^* cerebella compared with *Mcoln1^+/+^* cerebella. Therefore, the impact of *Mcoln1*/TRPML1 loss on oligodendrocyte maturation and myelination is brain-region specific; this should be taken into consideration when discussing the impact of MLIV and its treatment.

### Transcriptional analysis of the iron-regulated genes failed to reveal signs of iron depletion in the whole-cortical homogenates

Iron handling by the lysosomes has been shown to be impaired in *Mcoln1*/TRPML1-deficient cells and brain tissues (Coblentz et al., 2014; Dong et al., 2008; Grishchuk et al., 2015). Despite the existing evidence for iron involvement, the mechanism by which iron mishandling impacts *Mcoln1*/TRPML1-deficient cells is unclear. Iron uptake in many cell types is facilitated through receptor-mediated endocytosis and therefore its late events are handled by the endolysosomes (Todorich et al., 2009, 2011; Valerio, 2007). TRPML1 may be involved in this by transferring ferrous iron from the lysosomal lumen to the cytoplasm. It is, therefore, possible that *Mcoln1*/TRPML1 loss traps ferrous iron in the lysosomes, depriving the cytoplasm of iron and causing lysosomal oxidative stress. Importantly, cytoplasmic iron is key to oligodendrocyte maturation and myelin production as it is a co-factor in ATP production, which is highly important due to extremely high energy requirements of oligodendrocytes during myelination, and in the activity of key enzymes of lipid catabolism and biosynthesis required for myelin production (Todorich et al., 2009). Iron retention in the lysosome due to loss of *Mcoln1*/TRPML1 might therefore directly affect oligodendrocyte maturation by depriving the reactions of iron.

**Fig. 5.**
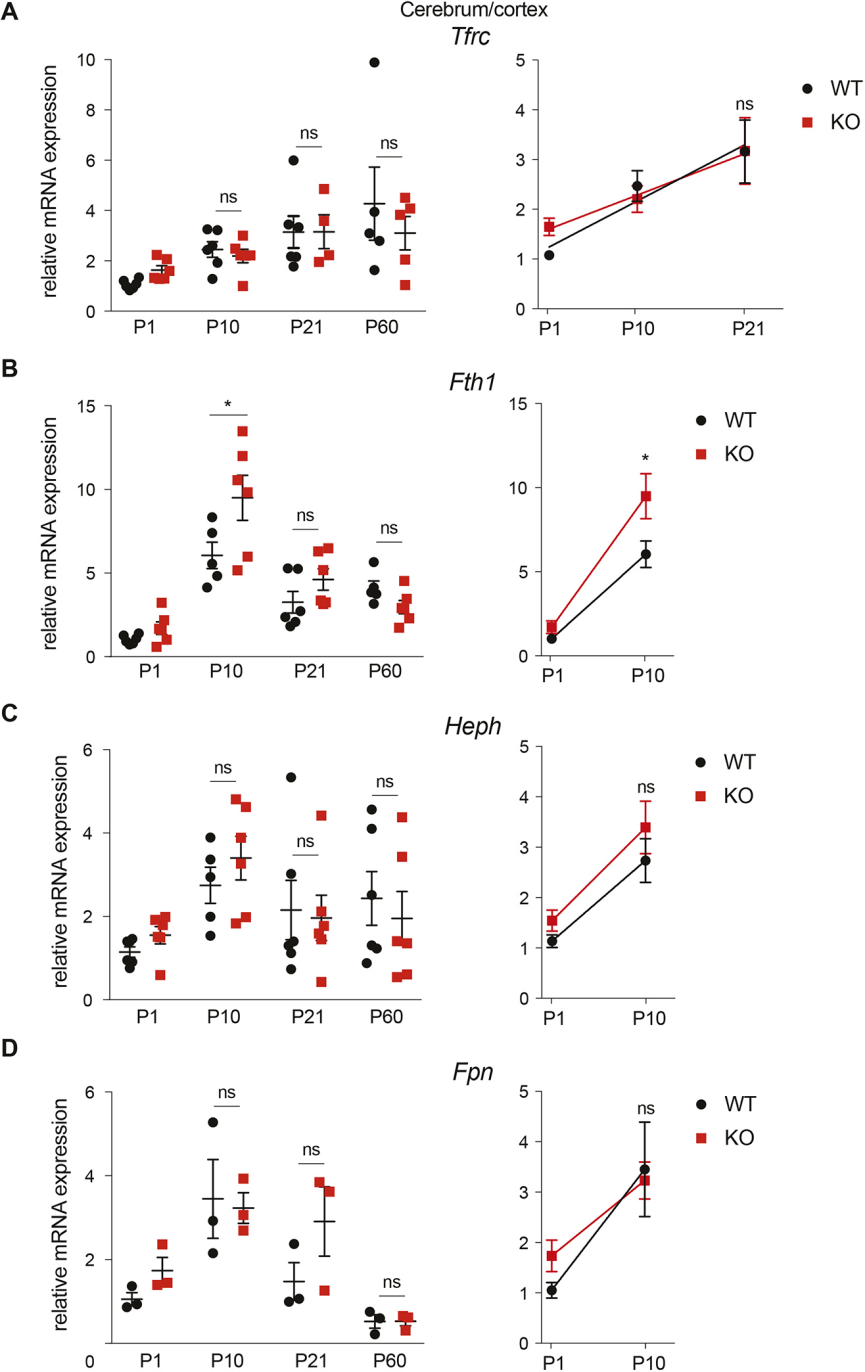
**Cytoplasmic iron markers in the MLIV mouse forebrain.** (A-D) qPCR analysis of mRNA for cytoplasmic iron markers *Tfrc* (A), *Heph* (B), *Fth1* (C) and *Fpn* (D) in the cerebrum (P1) and cortex (P10-P60) samples of WT (*Mcoln1*^+/+^) and *Mcoln1*^−/−^ (KO) mice. The analysis was performed using the ΔΔ^Ct^ method; *Actb* was used as a housekeeping gene. *P<0.05 using two-way ANOVA followed by Tukey multiple comparison test; *n*=3-5; ns, *P*>0.05; error bars represent s.e.m.

To answer whether *Mcoln1*/TRPML1 loss is associated with the depletion of the cytoplasmic iron, we analyzed the expression of the iron-regulated genes, such as transferrin receptor (*Tfrc*), hephaestin (*Heph*), ferritin heavy chain 1 (*Fth1*) and ferroportin (*Fpn*; also known as *Slc40a1*), by qPCR. All these genes are regulated by cytoplastic iron through Hif1α, Hif2α and other transcription factors involved in metal handling and oxidative stress (Acikyol et al., 2013; Li et al., 2013). qPCR showed no distinct difference between the WT and *Mcoln1^−/−^* samples (Fig. 5). We, therefore, conclude that whole-tissue transcriptomic analysis reports no brain-wide cytoplasmic iron depletion. Owing to the relatively low abundance of oligodendroglial mRNA in the whole-tissue samples, however, we cannot rule out whether dysregulation of these genes in *Mcoln1^−/−^* animals takes place only in oligodendrocytes and may be masked by the signal from more-abundant cell types.

## DISCUSSION

In the present study, we show that loss of the lysosomal channel TRPML1 is associated with delayed oligodendrocyte maturation leading to brain hypomyelination, which is a characteristic of both the MLIV mouse model and the human disease. A dramatically significant aspect of MLIV uncovered by these studies is the extremely early onset of the oligodendrocyte maturation delay, manifesting already by P10. Importantly, these early myelination deficits are not reflecting or causing underlying structural abnormalities of neuronal fibers or their loss. This is a key piece of information for designing MLIV treatments.

We have previously reported reduced myelination in *Mcoln1^−/−^* mice via expression analysis of oligodendrocyte markers MAG, MBP, MOBP and platelet-derived growth factor receptor (PDGFR; also known as PDGFRB) in whole-brain homogenates at 10 days, 2 months and 7 months of age (Grishchuk et al., 2015). We also showed lower protein levels of the mature oligodendrocyte marker PLP1 in the *Mcoln1*^−/−^ brain and reduced thickness of the PLP1-immunostained corpus callosum. Importantly, the neurofilament marker SMI312 remained unchanged in the early symptomatic *Mcoln1^−/−^* mice (2 months of age), showing normally formed neuronal fibers in the corpus callosum. In the present study, we tracked and compared oligodendrocyte maturation markers in the forebrain/cortex and cerebellum in *Mcoln1^−/−^* mice at 1, 10, 21 and 60 days after birth. We found dramatic differences between genotypes and brain regions. More specifically, while we report significant myelination deficits in the anterior regions (cortex, corpus callosum) of the *Mcoln1^−/−^* brain, myelination in the posterior brain (cerebellum) was normal. This regional and temporal pattern of myelination deficits in the *Mcoln1^−/−^* mouse, along with the time course of *Mcoln1/*TRPML1 expression in postnatal brain development, provides new insights into the role of the TRPML1 channel in the myelination process. It is well recognized that myelination progresses from caudal to rostral pathways and in the order of increasing complexity of brain function (Gerber et al., 2010; Purger et al., 2016), starting from the areas responsible for basic homeostatic functions (pons, cerebellum) and proceeding to the areas controlling more complex tasks (frontal cortex). In the mouse, deposition of myelin in the cerebellum starts in the deepest regions earlier than P6 and is largely accomplished by P23 (Purger et al., 2016). In the corpus callosum, the first myelinating cells are noticed on P9. In TRPML1-deficient brains, the regions that normally acquire myelination later in development are affected the most. Interestingly, we show that, in parallel with brain myelination, expression of TRPML1 steadily increases in the postnatal brain and is more prominent in the cortex at the final stage of the myelination process, indicating its need for myelination of the higher-hierarchical regions.

In corroboration with our findings in *Mcoln1^−/−^* KO mice, specific abnormalities in cerebral white matter have been observed in MLIV patients. In a cross-sectional cohort analysis of 15 MLIV patients ranging in age from 16 months to 22 years (Frei et al., 1998), hypoplasia of the corpus callosum and elevated T2 signal in the subcortical white matter were reported in all patients, while cortical atrophy was only noted in two of the oldest (15 and 22 years). A subsequent prospective study of brain magnetic resonance imaging (MRI) changes in five MLIV patients, ranging from 7 to 18 years of age, found increasing cortical gray matter and decreasing subcortical white-matter volumes across a 2- to 3-year follow-up period (Schiffmann et al., 2014). Although additional longitudinal data in individual patients are still needed to support conclusions, the available data in humans illustrate an early developmental defect followed by a late degenerative process specifically affecting white-matter tracts. The possibility that axonal degeneration could accompany white matter loss in MLIV patients was raised by the finding of decreased N-acetylaspartate (NAA)/creatine or choline ratios in multiple cortical regions (Bonavita et al., 2003; Cambron et al., 2012). Decreased NAA levels, however, can be seen in the setting of neuronal mitochondrial dysfunction without neuronal degeneration. Taken together with our current findings, we propose that the neurological deficits observed in the *Mcoln1* KO mice and MLIV patients mainly arise from abnormalities in CNS white matter.

Our findings in *Mcoln1^−/−^* mice have important implications for understanding the disease pathogenesis in MLIV patients, for whom data on the earliest stages of myelination are not available. In regards to myelination, P10 in mice is roughly equivalent to the full-term human neonate (Hagberg et al., 2002; Semple et al., 2013). While corpus callosum hypoplasia/dysgenesis and subcortical white-matter abnormalities have consistently been reported in older individuals (Frei et al., 1998), brain MRI data for MLIV patients younger than 16 months of age have not been reported, largely due to the first symptoms presenting around 6 months of age and diagnosis rarely occurring before the first year. The delayed manifestation of motoric dysfunction in MLIV patients might reflect selective myelination deficit in higher-cortical regions. This is also supported by the fact that in the first months of life (prior to MLIV symptom onset) motor function is mostly attributable to primitive brain regions, where, based on our mouse data, myelination is not affected by the loss of *Mcoln1/*TRPML1.

Our understanding of leukodystrophies, genetically determined disorders that selectively affect white matter in the CNS, has rapidly evolved with the recent application of brain imaging techniques paired with next-generation sequencing (van der Knaap and Bugiani, 2017). Surprisingly, mutations in myelin- or oligodendrocyte-specific proteins are only responsible for a portion of clinically discernible leukodystrophies, while genes important for housekeeping processes in multiple cell linages are largely responsible for the remainder. As a result, the new extended classification of the leukodystrophies now includes more than 50 genetic syndromes with mutations in myelin- or oligodendrocyte-specific genes (hypomyelinating and demyelinating leukodystrophies; leukodystrophies with myelin vacuolization), and other white-matter components including axons (leuko-axonopathies), glia (astrocytopathies and microgliopathies) and blood vessels (leukovasculopathies). We believe that the prominent and early involvement of developmental hypomyelination strongly advocates for MLIV to be included to this list.

At the cellular level, TRPML1 has been shown to be involved in various cell functions including membrane fission and fusion, as well as the redistribution of ions between the lysosomes and the cytoplasm (Cheng et al., 2014; Dong et al., 2008; Eichelsdoerfer et al., 2010; Kukic et al., 2013; Miao et al., 2015; Samie et al., 2013; Soyombo et al., 2006; Venkatachalam et al., 2008; Wong et al., 2012; Xu and Ren, 2015; Xu et al., 2006; Zhang et al., 2012). The latter effects were or can be attributed to TRPML1 iron conductance, or its role in the traffic of iron or iron-bound proteins in the endocytic pathway. Impairment of TRPML1-dependent iron uptake is an attractive hypothesis for the oligodendrocyte maturation deficits in the MLIV brain, as cytoplasmic iron is a key co-factor of the myelin synthesis pathway (Todorich et al., 2009). Indeed, iron deficiency and abnormal cellular iron absorption are associated with hypomyelination (Morath and Mayer-Pröschel, 2001; Todorich et al., 2009, 2011). Iron handling deficits have been reported by us in the brain of the *Mcoln1*^−/−^ mouse model (Grishchuk et al., 2015). At that time, we were unable to provide a definitive answer regarding the impact of iron mishandling in the *Mcoln1*^−/−^ brain. Here, we extended our studies using a battery of iron-reporter genes but found no evidence of the cytoplasmic iron deficit in the *Mcoln1^−/−^* cortical tissue homogenates. We, therefore, conclude that iron mishandling caused by *Mcoln1*/TRPML1 loss is either (1) limited to a specific subset of cells (such as mature oligodendrocytes) and being masked in the whole-tissue preparations, (2) taking place at certain developmental stages, and/or (3) only manifesting as a lysosomal iron buildup without significantly affecting the cytoplasmic iron uptake and therefore unable to be detected via analysis of expression of the iron-related genes. To this end, isolated *Mcoln1^−/−^* oligodendrocyte cultures and co-cultures with neurons and glia will be instrumental in delineating the mechanism by directly measuring cytosolic iron reporters, lysosomal iron content, oxidative stress and their impact on myelination, and will be the focus of our future study.

In conclusion, we present evidence of the role of the TRPML1 lysosomal channel in developmental myelination. Genetic ablation of TRPML1 leads to deficient oligodendrocyte maturation, resulting in hypomyelination of the cortex and corpus callosum already noticeable at P10. However, mechanisms of how the loss of TRPML1 leads to such deficits remain unknown. Brain myelination in development is a complex and highly regulated process that depends on both cell-intrinsic (oligodendroglial) and -extrinsic (neuronal, astrocytic and microglial) signaling and cues. Indeed, reduced myelination in the early-postnatal *Mcoln1^−/−^* cortex is accompanied by the observations of robust activation of astrocytes, resulting in elevated cytokine/chemokine release and neuroinflammation (Weinstock et al., 2018). Therefore, better understanding of the role of TRPML1 in oligodendrocyte maturation and myelin deposition will require new genetic *in vivo* (using the Cre-LoxP system for cell-type-specific manipulation of *Mcoln1* expression) and *in vitro* oligodendrocyte and other brain cell co-culture models and this will be the scope of our future investigations. Elucidating the involvement of other brain cell types, neurons and glia, affected by the loss of TRPML1 in the development of the oligodendrocyte lineage cells is important, given the complex picture of brain pathology and dysfunction in this disease.

## MATERIALS AND METHODS

### Animals

*Mcoln1* KO mice were maintained and genotyped as previously described (Venugopal et al., 2007). The *Mcoln1^+/−^* breeders for this study were obtained by backcrossing onto a C57Bl6J background for more than ten generations. *Mcoln1^+/+^* littermates were used as controls. Experiments were performed according to the institutional and National Institutes of Health guidelines and approved by the Massachusetts General Hospital Institutional Animal Care and Use Committee.

### Immunohistochemistry and image analysis

To obtain brain tissue for histological examination 1-, 10-, 21- and 60-day-old *Mcoln1^−/−^* and control mice were sacrificed using a carbon dioxide chamber. Immediately after, they were transcardially perfused with ice-cold phosphate-buffered saline (PBS). Brains were post-fixed in 4% paraformaldehyde in PBS for 48 h, washed with PBS, cryoprotected in 30% sucrose in PBS for 24 h, split into cerebrum and cerebellum, frozen in isopentane and stored at −80°C. Brains were bisected along the midline and one hemisphere was examined histologically. Coronal sections of the cerebrum and sagittal sections of the cerebellum were cut at 10 μm using a cryostat. Sections were mounted onto glass SuperFrost plus slides. These sections were stored at −20°C before any staining procedures. For PLP1 and SMI312 staining, sections were microwaved in citrate buffer (10 mM citric acid, 0.05% Tween 20, pH 6.0) at low power for 18 min for antigen retrieval, blocked in 0.5% Triton X-100 and 5% normal goat serum (NGS) in PBS and incubated with primary antibodies diluted in 1% NGS overnight. The following primary antibodies were used: PLP1 rabbit polyclonal (1:500; ab28486) and SMI312 (pan axonal neurofilament) mouse monoclonal (1:1000; ab24574), both from Abcam (Cambridge, MA, USA). Sections were incubated with secondary antibodies in 1% NGS for 1.5 h at room temperature. The following secondary antibodies were used: donkey anti-mouse AlexaFluor 488 and donkey anti-rabbit AlexaFluor 555 (1:500; Invitrogen, Eugene, OR, USA). For APC-CC1 staining no antigen retrieval was performed. After blocking, sections were incubated with anti-APC-CC1 antibody (mouse monoclonal; 1:100; OP80, Calbiochem, Billerica, MA, USA), followed by goat anti-mouse AlexaFluor 488 (1:500; Invitrogen). Sections were counterstained with NucBlue nuclear stain (Life Technologies, Eugene, OR, USA).

Images were acquired on DM8i Leica Inverted Epifluorescence Microscope with Adaptive Focus (Leica Microsystems, Buffalo Grove, IL, USA) with Hamamatsu Flash 4.0 camera and advanced acquisition software package MetaMorph 4.2 (Molecular Devices, San Jose, CA, USA) using an automated stitching function. The exposure time was kept constant for all sections (WT and *Mcoln1^−/−^* sections within the same immunohistochemistry experiment). Image analysis was performed using Fiji software (National Institutes of Health, Bethesda, MD, USA). Areas of interest were selected in each section. Area and mean pixel intensity values were compared between genotypes using GraphPad Prism 7.04 software (GraphPad, La Jolla, CA, USA) using unpaired Student's *t*-test. Gaussian distribution was tested using the Shapiro–Wilk normality test.

### RNA extraction and qRT-PCR

For qRT-PCR experiments, total RNA was extracted from whole cerebra or dissected cerebella from P1 pups. Whole-cerebella and cortical samples from the brains of 10-day-, 21-day- and 2-month-old mice were used. Cortical samples were dissected to include motor and somatosensory areas and did not contain corpus callosum tissue. RNA was extracted using TRizol (Invitrogen, Carlsbad, CA, USA), according to the manufacturer's protocol. All experimental groups were designed to contain at least four animals per genotype and time point; lower numbers in some conditions were due to low mRNA yields that prevented them being included in qRT-PCR experiments. For complementary DNA (cDNA) synthesis, MuLV Reverse Transcriptase (Applied Biosystems, Foster City, CA, USA) was used with 2 μg total RNA and oligo(dT)18 (IDT, Coralville, IA, USA) as a primer. qRT-PCR was carried out using 1:75 dilutions of cDNA, 2× SYBR Green/ROX Master Mix (Fermentas, Glen Burnie, MD, USA) and 4 μM primer mix per 10 μl reaction. For gene expression analysis, the following primers were used (IDT, Coralville, IA, USA): *Actb*, forward 5′-GCTCCGGCATGTGCAAAG-3′ and reverse 5′-CATCACACCCTGGTGCCTA-3′; *Fpn*, forward 5′-ATCCTCTGCGGAATCATCCT-3′ and reverse 5′-CAGACAGTAAGGACCCATCCA-3′; *Fth1*, forward 5′-TGATCCCCACTTATGTGACTTCAT-3′ and reverse 5′-ACGTGGTCACCCAGTTCTTT-3′; *Heph*, forward 5′-ACTAAAGAGATTGGAAAAGCAGTGA-3′ and reverse 5′-TCATGCCCAGCATCTTCACA-3′; *Mag*, forward 5′-CGGGATTGTCACTGAGAGC-3′ and reverse 5′-AGGTCCAGTTCTGGGGATTC-3′; *Mbp*, forward 5′-GTGCCACATGTACAAGGACT-3′ and reverse 5′-TGGGTTTTCATCTTGGGTCC-3′; *Mobp*, forward 5′-CACCCTTCACCTTCCTCAAC-3′ and reverse 5′-TTCTGGTAAAAGCAACCGCT-3′; *Ng2*, forward 5′-CATTTCTTCCGAGTGGTGGC-3′ and reverse 5′-CACAGACTCTGGACAGACGG-3′; *Tfrc*, forward 5′- GAGGCGCTTCCTAGTACTCC-3′ and reverse 5′-TGGTTCCCCACCAAACAAGT-3′.

To avoid amplification of genomic DNA and ensure amplification of cDNA, all primers were designed to span exons and negative reverse transcription reactions (without reverse transcriptase) were performed as a control. Samples were amplified in triplicate on a 7300 Real-Time System (Applied Biosystems, Foster City, CA, USA) using the following program: 2 min at 50°C, 10 min at 95°C, and 40 cycles at 95°C for 15 s followed by 1 min at 60°C. In addition, a dissociation curve step was run to corroborate that amplification with specific primers resulting in one product only. The ΔΔ^Ct^ method was used to calculate relative gene expression, where Ct corresponds to the cycle threshold. Ct values for a representative set of genes are plotted in Fig. S2. ΔCt values were calculated as the difference between Ct values from the target gene and the housekeeping gene *Actb*. Experiments were performed double blind using numerically coded samples; the sample identity was disclosed at the final stage of data analysis. Data are presented as fold change.

## Supplementary Material

Supplementary information
