# Supplementary material for: Early evidence of delayed oligodendrocyte maturation in the mouse model of mucolipidosis type IV
Source: Dis Model Mech. 2020 Jul 30;13(7):dmm044230. doi: 10.1242/dmm.044230 (PMC7406328; doi:10.1242/dmm.044230)
Supplement: Supplementary information [file dmm-13-044230-s1.pdf]

Supplementary Figure 1

A

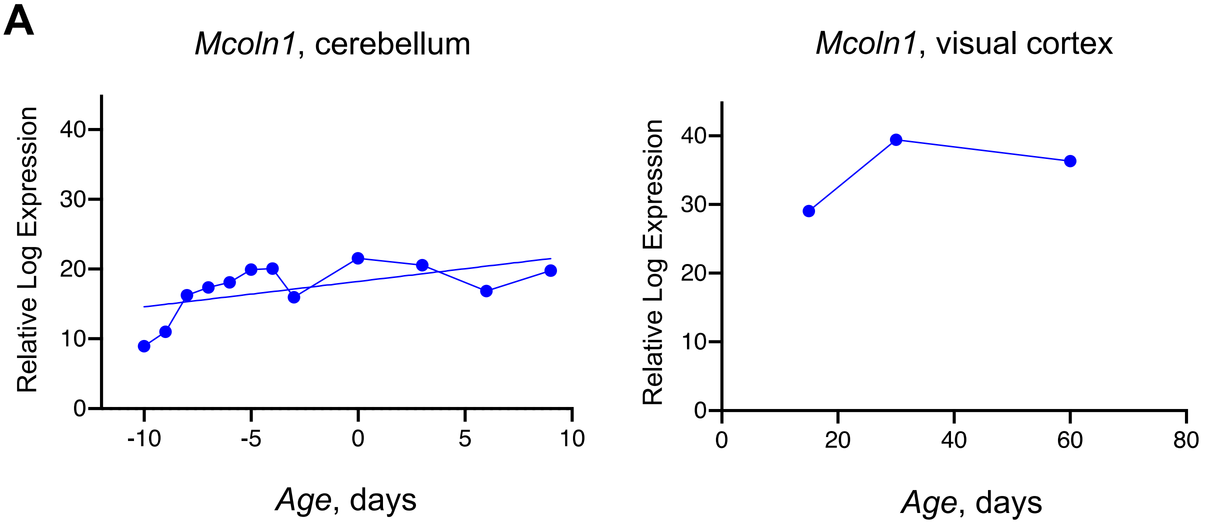

B

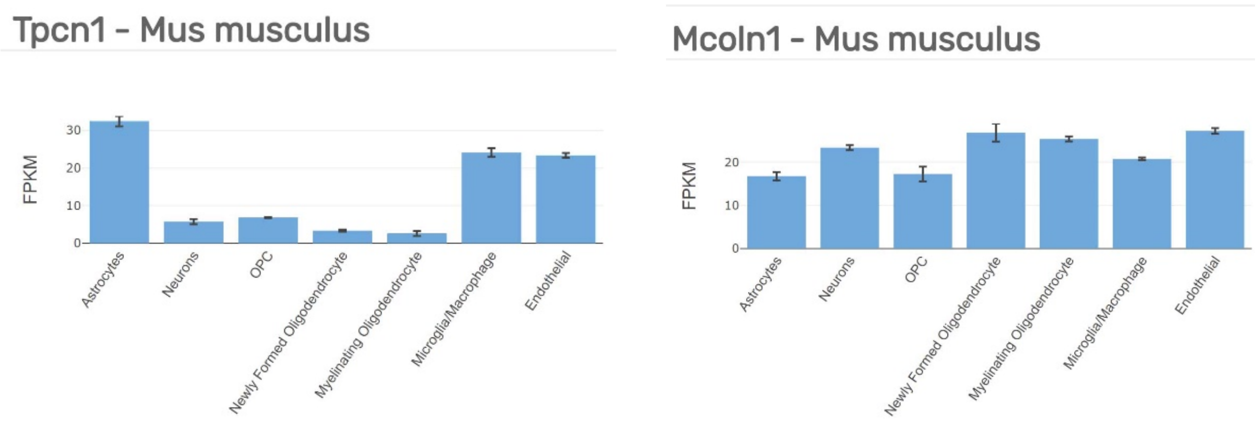

Fig S1. (A) *Mcoln1* expression in the mouse cerebellum or visual cortex from FANTOM (Functional ANnoTation Of Mammalian Genome) data set. Expression levels are shown as the average of (relative log expression) RLE normalized TPM counts; n = 3-4 for each time-point. Visual cortex values obtained from (Vitezic et al., 2014) and cerebellum data from (Ha et al., 2019). All data have been extracted using the FANTOM5 Table Extraction Tool. (B) Cell-type specific expression of either *Mcoln1* or *Tpcn1* in the mouse brain from (Zhang et al., 2014);

Disease Models & Mechanisms • Supplementary information

Supplementary Figure 2

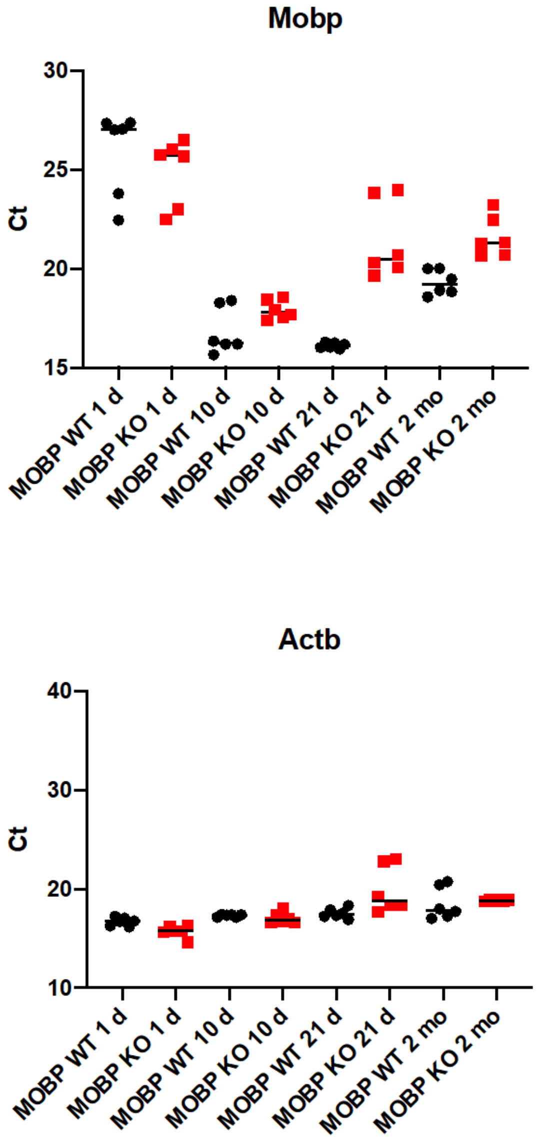

Fig S2. Sample Ct values obtained using qPCR with *Mobp* and *Actb* primers. Vertical axis: individual Ct values.
